# A curated catalog of canine and equine keratin genes

**DOI:** 10.1371/journal.pone.0180359

**Published:** 2017-08-28

**Authors:** Pierre Balmer, Anina Bauer, Shashikant Pujar, Kelly M. McGarvey, Monika Welle, Arnaud Galichet, Eliane J. Müller, Kim D. Pruitt, Tosso Leeb, Vidhya Jagannathan

**Affiliations:** 1 Division of Clinical Dermatology, Department of Clinical Veterinary Medicine, Vetsuisse Faculty, University of Bern, Bern, Switzerland; 2 Dermfocus, Vetsuisse Faculty, University of Bern, Bern, Switzerland; 3 Institute of Genetics, Vetsuisse Faculty, University of Bern,Bern, Switzerland; 4 National Center for Biotechnology Information, National Library of Medicine, National Institutes of Health, Bethesda, MD, United States of America; 5 Institute of Animal Pathology, Vetsuisse Faculty, University of Bern, Bern, Switzerland; 6 Department of Clinical Research, Molecular Dermatology and Stem Cell Research, University of Bern, Bern, Switzerland; 7 Clinic for Dermatology, Inselspital, Bern University Hospital, Bern, Switzerland; University of Sydney Faculty of Veterinary Science, AUSTRALIA

## Abstract

Keratins represent a large protein family with essential structural and functional roles in epithelial cells of skin, hair follicles, and other organs. During evolution the genes encoding keratins have undergone multiple rounds of duplication and humans have two clusters with a total of 55 functional keratin genes in their genomes. Due to the high similarity between different keratin paralogs and species-specific differences in gene content, the currently available keratin gene annotation in species with draft genome assemblies such as dog and horse is still imperfect. We compared the National Center for Biotechnology Information (NCBI) (dog annotation release 103, horse annotation release 101) and Ensembl (release 87) gene predictions for the canine and equine keratin gene clusters to RNA-seq data that were generated from adult skin of five dogs and two horses and from adult hair follicle tissue of one dog. Taking into consideration the knowledge on the conserved exon/intron structure of keratin genes, we annotated 61 putatively functional keratin genes in both the dog and horse, respectively. Subsequently, curators in the RefSeq group at NCBI reviewed their annotation of keratin genes in the dog and horse genomes (Annotation Release 104 and Annotation Release 102, respectively) and updated annotation and gene nomenclature of several keratin genes. The updates are now available in the NCBI Gene database (https://www.ncbi.nlm.nih.gov/gene).

## Introduction

Keratins are intermediate filament proteins of the epithelial cytoskeleton. They are expressed in a cell-, tissue- and differentiation-dependent manner in stratified (e.g. epidermis and cornea) and simple (liver, pancreas and intestine) epithelia as well as in skin appendages such a hairs and nails [1,2]. As structural proteins, keratins provide mechanical stability to maintain epithelial integrity and barrier function [3]. This is best exemplified in the epidermis. Keratins represent a major protein fraction of the keratinocytes, the main cell type in the epidermis. In the different layers of the epidermis, keratinocytes differentially express specific keratins, which correlate with their differentiation stage and contribute to the integrity of the epidermis through interaction with both cell-matrix and intercellular adhesion complexes [4,5]. Additionally, keratins are also involved in the regulation of cellular processes such as embryonic development as well as cell motility, proliferation and death by modulating signal molecule activity [6–11]. Furthermore, keratins were recently found to be major regulators of cellular stiffness, a key parameter in cancer development. Thus, the correct expression of specific keratin genes is essential for normal skin homeostasis [1].

Based on their isoelectric point, keratins can be grouped into type I (acidic) and type II (neutral or basic) keratins. Type I and type II keratins form heterodimers, which further assemble into intermediate filaments [12]. Keratins can also be grouped according to their specific expression pattern: 37 human keratins are specifically expressed in epithelia, while 17 are considered hair keratins. However, nine of the 37 epithelial keratins are also restricted to different compartments of the hair follicles [13].

The latest annotation of the human genome contains 55 functional keratin genes and so far, for most of them only a single transcript is known [14–16]. The human keratin genes are clustered at two different chromosomal locations. The first cluster on chromosome 17q21 contains all type I keratin encoding genes except for *KRT18*. The type II keratin encoding genes and *KRT18* are clustered on chromosome 12q13 [17]. Consistent with their important cellular functions, genetic variants in the keratin genes may cause abnormalities in skin, nails, hair and mucosa. Different genetic variants in at least 18 keratin genes have been found to be causative for human genodermatoses, hereditary diseases of the skin [18,19].

There is a high conservation of the keratin genes in mammals with respect to their organization in the genome, but also with respect to their conserved exon/intron structure suggesting multiple duplication events from an ancestral gene during evolution [20]. The mouse has 54 functional keratin genes, organized in two clusters on chromosomes 11 and 15, similar as in humans [17]. Dogs and horses have draft genome assemblies of relatively high quality, but their annotations are almost exclusively based on computational methods [21–24]. The high similarity between the numerous keratin genes as well as sequencing errors and gaps in the reference genome assembly make these predictions error prone. In the current dog and horse annotations there are examples, where exons from different keratin genes have been erroneously merged into computer-predicted keratin transcripts (e.g. Ensembl transcript ENSECAT00000023303 is composed of one exon of *KRT78* and six exons of *KRT8*).

Reliable keratin gene annotations are a requirement for research on heritable skin or hair disorders [25]. Therefore, the aim of this study was to present a curated catalog of canine and equine keratin genes. We based the curated annotation on evolutionary conserved structural features and homology to the corresponding human keratin genes complemented by canine and equine RNA-seq data. We also determined the genomic sequence of some critical gaps in the horse reference genome assembly to facilitate the correct annotation of keratin genes. NCBI RefSeq [26] curators then used our curated annotation and suggested nomenclature to guide updates to their annotation where possible.

## Methods

### Ethics statement

Animal work consisted of collecting blood samples and skin biopsies of privately owned dogs and horses with owners’ consent. All animal experiments were performed in accordance with the relevant local guidelines and approved by the committee for animal experiments of the canton of Bern (permits no. BE31/13 and BE75/16).

### Analyzed dataset, genome assemblies, and existing annotations

The human reference genome GRCh38.p2 assembly and the NCBI annotation release 107 were used for this investigation [14]. For the dog, we used the CanFam 3.1 assembly and the NCBI annotation release 103 (RefSeq release 75). For the horse, we used the EquCab 2.0 assembly and the NCBI annotation release 101 (RefSeq release 75). We also compared our data to the canine and equine annotation from Ensembl (release 87 for both species).

### NCBI annotation updates

This manuscript reflects a static comparison to dog annotation release 103 and horse annotation release 101. Since our initial analysis, the NCBI annotation was updated to dog annotation release 104 and horse annotation release 102 by routine employment of NCBI’s Eukaryotic Genome Annotation Pipeline (https://www.ncbi.nlm.nih.gov/genome/annotation_euk/process/). These pipeline updates resulted in improved agreement with our annotation, as several NCBI gene models that previously differed from our annotation were updated to models that match our annotation. Furthermore, RefSeq curators collaborated to perform a manual review of keratin gene annotation in dog and horse (dog annotation release 104 and horse annotation release 102), which resulted in additional updates. These updates include replacement of model RefSeqs (XM_, XP_, XR_) with known RefSeqs (NM_, NP_, NR_) and updates to gene names. NCBI has adopted most of our suggested nomenclature. For 21 of the dog keratin genes and 28 of the horse keratin genes the symbol was updated in RefSeq. (We have attached NIH publishing agreement as S3 File).

### Targeted sequencing of equine keratin gene fragments

We determined the sequence (accessions LT576418 and LT576419) of two genomic fragments from *KRT78* and *KRT85* that were missing from the equine reference genome assembly (chr6:69,933,880–69,934,077 and chr6:69459932–69460612 respectively). DNA from equine EDTA blood (sample FM2644 derived from a Franches-Montagnes horse) was isolated using the Nucleon Bacc2 kit (GE Healthcare Life Sciences) and these regions were PCR amplified using primers KRT78_F, TAAAGGAAAGGGTCCTGCAA and KRT78_R, GAGCGGGTCTCCAGAGATG or KRT85_F, TCTTCTTCTTGAAGCTTGACCTG and KRT85_R, ACACCCAGCACAGGCAGAC. PCR products were treated with shrimp alkaline phosphatase and exonuclease I and then sequenced on both strands using ABI v3.1 BigDye chemistry and an ABI3730 capillary sequencer.

### RNA-seq

We isolated total RNA from skin biopsies of 3 dogs (DS032, DS042, LA1666 (PRJEB14110 and PRJEB14109)), hair follicle tissue from 1 dog (PRJEB14110), and a skin biopsy from 1 horse (UKH004) (PRJEB12979) using the RNeasy Fibrous Tissue Mini kit (Qiagen). Prior to RNA extraction the tissue was mechanically disrupted using the TissueRuptor device (Qiagen). The RNA samples were transformed into illumina TruSeq libraries and 2 x 150 bp sequencing reads were obtained on a HiSeq3000 instrument (illumina) at the Next Generation Sequencing Platform, University of Bern. The reads were filtered for low quality bases using a Phred quality score threshold of 15 for each base and reads longer than 50 bases were retained. The star aligner [27] was used to map the quality filtered reads to Ensembl CanFam3.1 reference using recommended parameters ‘—outFilterType BySJout’ and ‘—outFilterMultimapNmax 20’. We additionally used publicly available illumine skin RNA-seq datasets from the skin of two dogs, a Beagle and a dog of unspecified breed [22] as well as from the skin biopsy of one domestic horse showing Leopard complex spotting (EBI accessions PRJNA78827 and PRJEB3095). The reads of the downloaded RNA-seq data sets were also filtered and mapped to the reference genome in the same manner as described above. Exon level coverage was calculated using BedTools [28].

### Whole genome sequence data

83 paired end whole genome sequences from 28 diverse breeds of horses were mapped to the reference genome (NCBI) EquCab2.0 using BWA-MEM (version 0.7.10). The aligned reads were further coordinate sorted with samtools 1.2 [29] and duplicate marked with picard tools (http://broadinstitute.github.io/picard/). The whole genome datasets are available publicly at the European Nucleotide Archive (ENA; study accessions PRJEB14779, PRJEB5942, PRJEB10098, PRJEB12984, PRJEB12979, PRJEB9269, PRJEB9267, PRJEB9139, PRJEB14651).

### Comparison of RefSeq transcripts to RNA-seq data

Keratin gene clusters in the canine and equine genomes were identified by searching the NCBI Gene database for keratin genes corresponding to the first and last keratin gene of the orthologous human clusters. The identified chromosomal regions were inspected using Genome Data Viewer [30]. All the NCBI RefSeq transcripts in the cluster regions of the human, dog and horse genomes were downloaded from NCBI (S1 Table). If a keyword search with the human gene symbol did not lead to the corresponding horse or dog keratin gene, the genomic sequence of the human keratin gene of interest was used as query in a BLASTN search against the genome of the respective species. BLASTN hits and the relative position of the potential dog or horse keratin gene within the cluster compared to the relative position within the human keratin gene cluster were then used to identify putatively orthologous keratin genes.

Untranslated regions (UTRs), start and stop codons of the coding sequence as well as exon-intron boundaries of the RefSeq transcripts were visually compared to RNA-seq data from dog skin and dog hair follicles as well as from horse skin using the Integrative Genome Viewer (IGV) [31]. In case the RNA-Seq data did not support a coordinate of the RefSeq transcript annotation and if there was clear evidence from the RNA-Seq data for different exon coordinates, the RNA-seq supported exon coordinates were adopted for the curated annotation, which in case of equine *KRT9* and *KRT41* identified frameshifts of the conserved open reading frames in the genomic reference sequence.

## Results

### Genomic organization of keratin gene clusters

**Fig 1:**
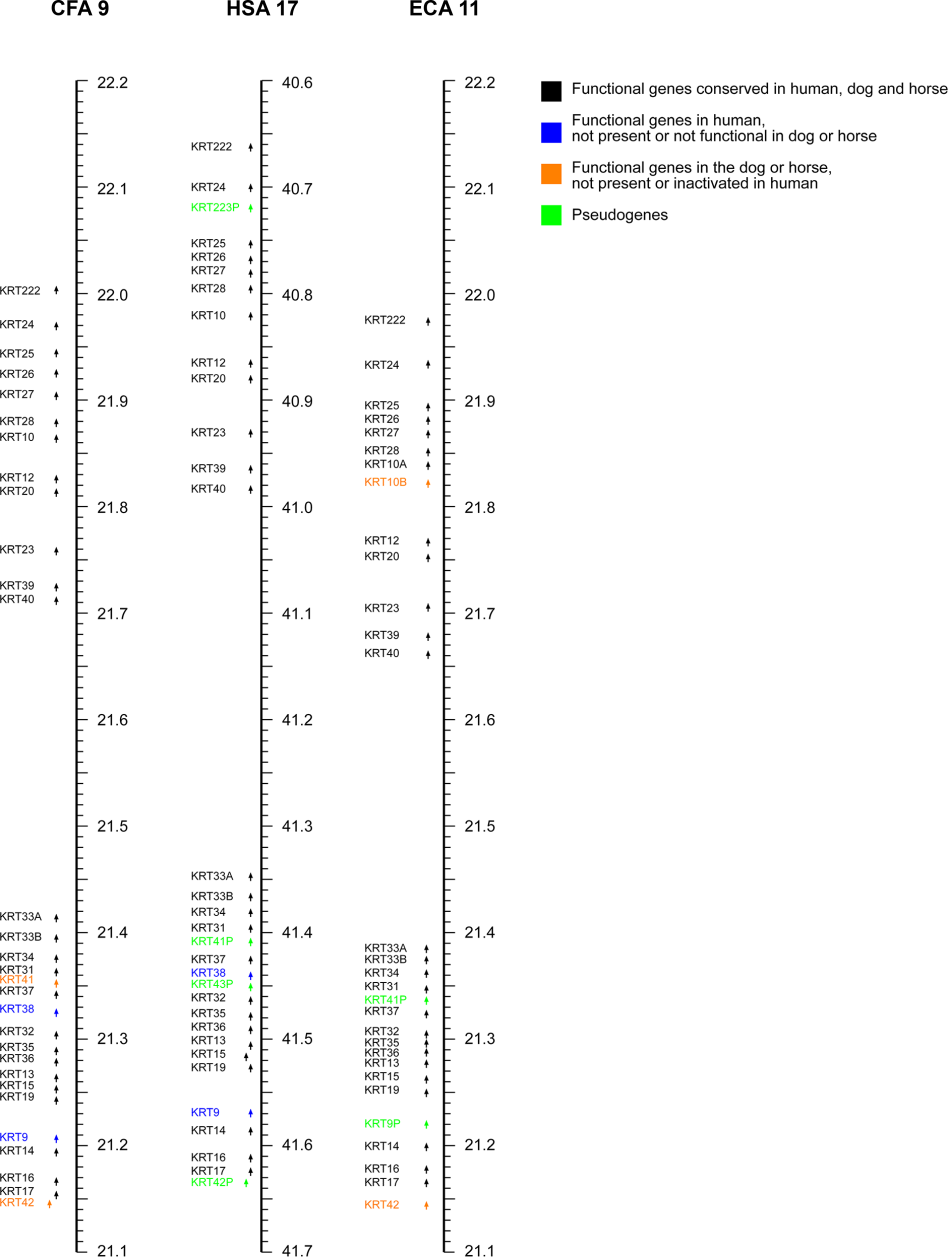
Comparative map of the keratin type I gene cluster. Type I keratin genes except *KRT18* in the dog, human and horse genomes. Arrows indicate the orientation of the genes. Note that CFA 9 and ECA 11 are represented in reverse orientation with decreasing coordinates from top to bottom. The genes’ nomenclature in the figure corresponds to our updated nomenclature for keratin genes (see Methods). S3 Table details the correspondence of gene symbols to NCBI Gene IDs and RefSeq IDs.

Type I keratin genes except for *KRT18* are clustered on human chromosome 17 (HSA 17), the corresponding gene clusters in dogs and horses are located on chromosomes 9 and 11 (CFA 9 and ECA 11). Compared to the human cluster, the keratin type I gene cluster is in reverse orientation in both dog and horse (Fig 1; S1 Table; S2 Table).

There are 28 functional keratin genes and four pseudogenes (gene names with suffix P) in the human keratin type I gene cluster on HSA 17. Many 1:1 orthology relationships between the human, canine and equine genes exist and the synteny is largely conserved. However, in dogs, the homologs of two human pseudogenes appear to be still active and functional (*KRT41P* and *KRT42P*). Transcription of both genes is supported by the available RNA-seq data (average exon coverage of 21x and 161x respectively). We suggest the gene symbols *KRT41* and *KRT42* for the corresponding canine genes. Homologs for the other two human pseudogenes *KRT43P* and *KRT223P* are missing in the dog reference genome assembly. Thus the canine keratin type I gene cluster most likely contains 30 functional genes.

**Fig 2:**
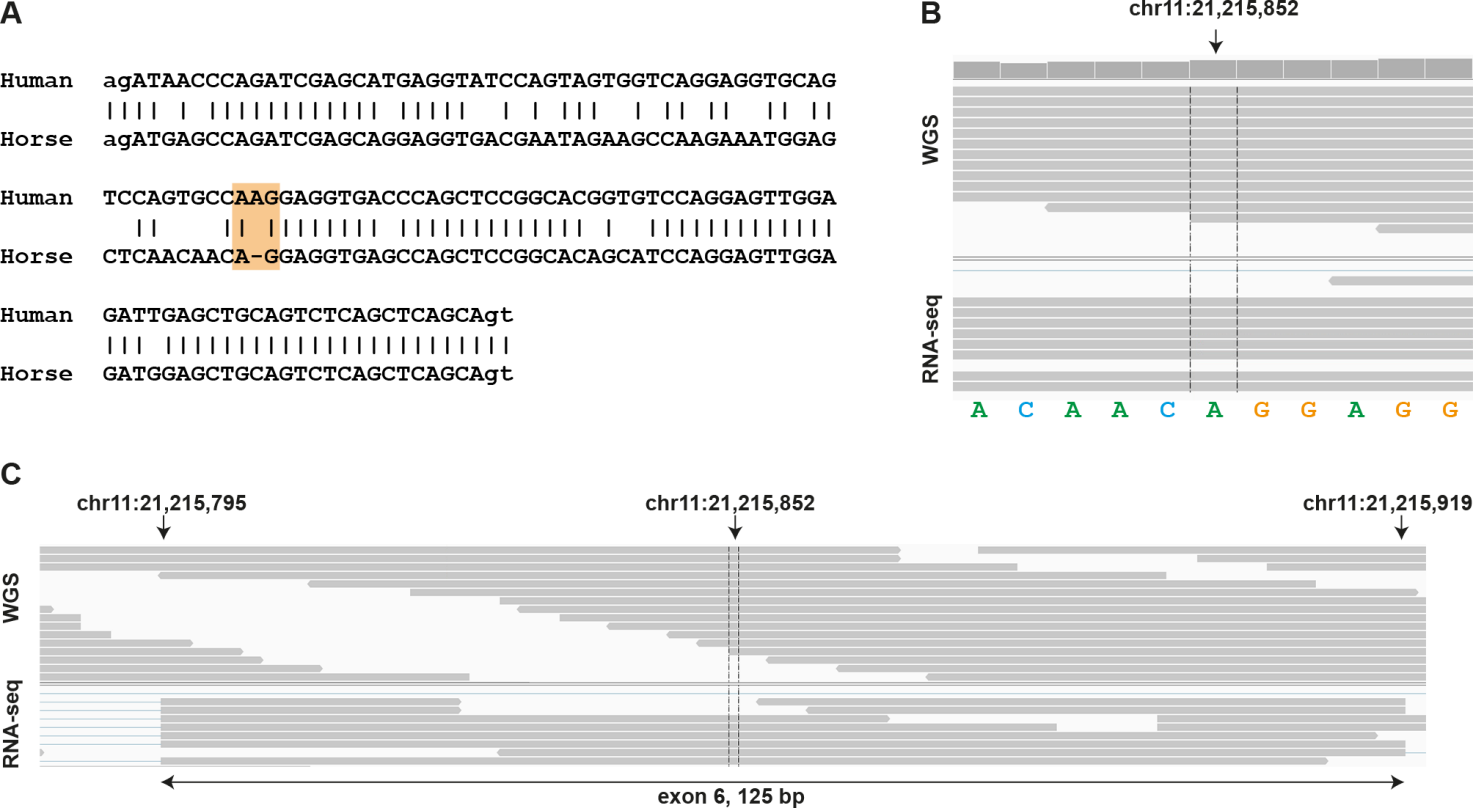
Frameshift deletion in exon 6 of the equine *KRT9* gene. A) Exon 6 alignment of equine *KRT9P* against the human ortholog *KRT9*. The yellow block shows the deletion of a single base in the horse exon leading to a frameshift. B) Illumina whole genome sequence and RNA-seq alignment confirms that the equine reference sequence does not contain a sequencing error which supports the claim of deletion. C. Expanded alignment region containing the frameshift deletion.

In the horse there has been a duplication event of the *KRT10* gene with respect to humans and dogs (S1 Fig). We suggest the gene symbols *KRT10A* and *KRT10B* for the corresponding equine paralogs. On the other hand, there is no equine ortholog of the human and canine *KRT38* gene in the current horse genome assembly. *KRT42* is expressed, contains an intact open reading frame, and thus appears to be functional in horse, similar to dogs and differing from humans. The equine homolog of the human *KRT9* genes contains a 1 bp deletion in exon 6 compared to humans and most other mammals. The deletion leads to a frameshift and an early premature stop codon only two codons after the deletion. The genome reference and whole genome sequences from 83 genetically diverse horses are all in agreement without any evidence for intraspecies variation at the position of this deletion. RNA-seq also confirmed the presence of the 1 bp deletion in the equine homolog of the *KRT9* gene (Fig 2, S2 Fig). Thus the equine homolog of *KRT9* is most likely no longer functional. We therefore suggest the gene symbol *KRT9P* for the equine pseudogene. The equine homolog of human *KRT41P* contains a frameshift in the first exon and is thus also likely a pseudogene, for which we suggest the gene symbol *KRT41P*. There were no equine homologues of the remaining two human pseudogenes *KRT43P* and *KRT223P* and we did not find any evidence for transcribed pseudogenes or functional genes in these regions in our RNA-seq data. The orthologs of these pseudogenes might be missing from the horse reference genome assembly, similar to the situation in dogs. Thus, the equine keratin type I gene cluster most likely contains 28 functional genes and two pseudogenes (Table 1).

**Table 1:** Details of the genes in the keratin type I gene cluster on HSA 17, CFA 9, and ECA 11.

HSA 17 (GRCh38.p2)			CFA 9 (CanFam3.1)			ECA 11 (EquCab 2.0)		
Gene symbol	Exons	Encoded amino acids	Gene symbol^a^	Exons	Encoded amino acids	Gene symbol^a^	Exons	Encoded amino acids
*KRT9*	8	983	*KRT9*	8	674	*KRT9P*	(9)^b^	(P)^b^^,^^c^
*KRT10*	8	584	*KRT10*	8	568	*KRT10A*	8	559
*-*	-	-	*-*	-	-	*KRT10B*	8	596
*KRT12*	8	494	*KRT12*	8	507	*KRT12*	8	488
*KRT13*	8	458	*KRT13*	8	452	*KRT13*	8	451
*KRT14*	8	472	*KRT14*	8	482	*KRT14*	8	478
*KRT15*	8	456	*KRT15*	8	472	*KRT15*	8	469
*KRT16*	8	473	*KRT16*	8	477	*KRT16*	8	(472)^b^
*KRT17*	8	432	*KRT17*	8	433	*KRT17*	8	433
*KRT19*	6	400	*KRT19*	6	399	*KRT19*	6	411
*KRT20*	8	424	*KRT20*	8	434	*KRT20*	8	424
*KRT23*	8	422	*KRT23*	8	419	*KRT23*	8	428
*KRT24*	8	525	*KRT24*	8	541	*KRT24*	8	506
*KRT25*	8	450	*KRT25*	8	450	*KRT25*	8	450
*KRT26*	8	468	*KRT26*	8	464	*KRT26*	8	469
*KRT27*	8	459	*KRT27*	8	446	*KRT27*	8	460
*KRT28*	8	464	*KRT28*	8	464	*KRT28*	8	464
*KRT31*	7	416	*KRT31*	7	409	*KRT31*	7	416
*KRT32*	7	448	*KRT32*	7	448	*KRT32*	7	448
*KRT33A*	7	404	*KRT33A*	7	404	*KRT33A*	7	404
*KRT33B*	7	404	*KRT33B*	7	407	*KRT33B*	7	404
*KRT34*	7	436	*KRT34*	7	393	*KRT34*	7	393
*KRT35*	7	455	*KRT35*	7	455	*KRT35*	7	455
*KRT36*	7	467	*KRT36*	7	467	*KRT36*	7	461
*KRT37*	7	449	*KRT37*	7	440	*KRT37*	7	444
*KRT38*	7	456	*KRT38*	7	457	*-*	-	-
*KRT39*	7	491	*KRT39*	7	483	*KRT39*	7	492
*KRT40*	7	431	*KRT40*	7	431	*KRT40*	7	431
*KRT41P*	P^c^	P^c^	*KRT41*	7	437	*KRT41P*	7	(P)^b^^,^^c^
*KRT42P*	P^c^	P^c^	*KRT42*	10	(492)^b^	*KRT42*	8	453
*KRT43P*	P^c^	P^c^	*-*	-	-	*-*	-	-
*KRT222*	6	295	*KRT222*	6	295	*KRT222*	6	295
*KRT223P*	P^c^	P^c^	*-*	-	-	*-*	-	-

^a^ The gene symbols have been updated by NCBI Refseq curators. A comprehensive listing of gene symbols and NCBI Gene IDs is given in S3 Table.

^b^Data in brackets are of low reliability due to gaps in the genome reference assemblies and/or insufficient coverage in the canine and equine RNA-seq data.

^c^P indicates pseudogenes.

**Fig 3:**
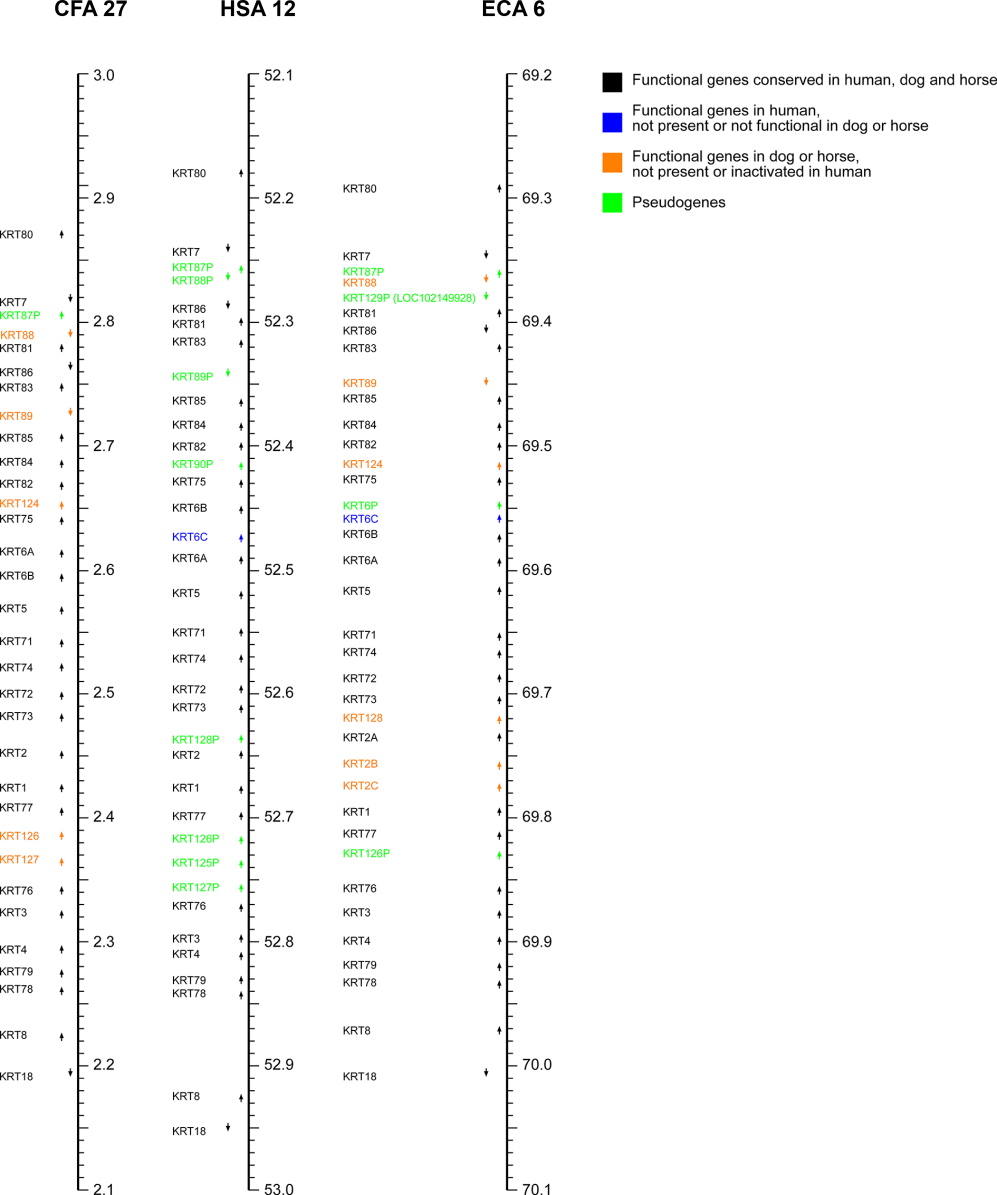
Comparative map of the keratin type II gene cluster. Type II keratin genes and *KRT18* in the dog (CanFam3.1), human (GRCh38.p2) and horse genomes (EquCab 2.0). Arrows indicate the orientation of the genes. Note that CFA 27 is represented in reverse orientation with decreasing coordinates from top to bottom. The genes’ nomenclature in the figure corresponds to our updated nomenclature for keratin genes (see Methods). S3 Table details the correspondence of gene symbols to NCBI Gene IDs and RefSeq IDs.

The keratin type II gene cluster located on HSA 12 in humans can be found on chromosomes 27 (CFA 27) and 6 (ECA 6) in dogs and horses. The canine gene cluster is in reverse orientation compared to human and horse (Fig 3; S1 Table).

There are 27 functional keratin genes and eight pseudogenes in the human keratin type II gene cluster. Similar to the keratin type I gene cluster, there are again many 1:1 orthology relationships between the human, canine and equine genes and the synteny is also largely conserved. In the canine cluster on CFA 27, the homologs of five human pseudogenes (*KRT88P*, *KRT89P*, *KRT90P*, *KRT126P*, *and KRT127P*) contain intact open reading frames and appear to be still functional (average exon level expression of 3.1x, 76.5x, 2.8x, 1024.3x and 71.1x, respectively). S4 Table lists descriptive summary statistics for the predicted canine proteins. Orthologs of the human *KRT6C* gene and the pseudogene *KRT128P* are missing from the dog reference genome assembly. The dog thus most likely has 31 functional genes and one pseudogene in the keratin type II gene cluster.

In the horse there have been duplication events involving the *KRT2* gene with respect to humans and dogs (S3 Fig). We suggest *KRT2A*, *KRT2B* and *KRT2C* as gene symbols for the corresponding equine paralogs. Five of the human pseudogenes appear to be functional in the horse based on intact open reading frames. *KRT6P* is an additional equine pseudogene that is missing from the dog and human reference genome assembly. On the other hand, there is no equine ortholog for the human *KRT125P* and *KRT127P*. The equine keratin gene with Gene ID: 102149928 (*LOC102149928)* is annotated as functional equine keratin gene with five exons. However, there is neither supporting evidence from our adult skin RNA-seq data for the expression of this gene, nor is there an ortholog in the human or dog reference genome assembly. Our suggested nomenclature is *KRT129P*, but this has not been accepted at NCBI due to an underlying gap (chr6:69,382,962–69,383,217) in the horse assembly. As the gene symbol *KRT124* has been proposed in the literature for the equine functional ortholog of human *KRT90P* [32], we suggest the gene symbol *KRT124* for the canine and equine genes (canine Gene ID: 486524, equine Gene ID: 100061458). Thus the equine keratin type II gene cluster most likely contains 33 functional genes and four pseudogenes (Table 2).

**Table 2:** Details of the genes in the keratin type II gene cluster on HSA 12, CFA 27, and ECA 6.

HSA 12 (GRCh38.p2)			CFA 27 (CanFam3.1)			ECA 6 (EquCab 2.0)			
Gene symbol	Exons	Encoded amino acids	Gene symbol^a^	Exons	Amino acids	Gene symbol^a^	Exons		Encoded amino acids
*KRT1*	9	644	*KRT1*	9	619	*KRT1*	9		626
*KRT2*	9	639	*KRT2*	9	635	*KRT2A*	9		629
-	-	-	*-*	-	-	*KRT2B*	9		618
-	-	-	*-*	-	-	*KRT2C*	(9)^b^		(618)^b^
*KRT3*	9	628	*KRT3 (LOC100683401)*	9	610	*KRT3*	9		630
*KRT4*	9	520	*KRT4*	9	530	*KRT4*	9		607
*KRT5*	9	590	*KRT5*	9	597	*KRT5*	9		593
*KRT6A*	9	564	*KRT6A (LOC486523)*	9	570	*KRT6A*	9		574
*KRT6B*	9	564	*KRT6B (LOC100687987)*	9	570	*KRT6B*	9		562
*KRT6C*	9	564	*-*	-	-	*KRT6C*	9		562
-	-	-	*-*	-	-	*KRT6P*	P^c^		P^c^
*KRT7*	9	469	*KRT7*	9	468	*KRT7*	9		465
*KRT8*	9	511	*KRT8*	9	491	*KRT8*	9		435
*KRT18*	7	430	*KRT18*	7	431	*KRT18*	6		430
*KRT71*	9	523	*KRT71*	9	525	*KRT71*	9		525
*KRT72*	9	511	*KRT72*	9	523	*KRT72*	9		523
*KRT73*	9	540	*KRT73*	9	589	*KRT73*	9		540
*KRT74*	9	529	*KRT74*	9	533	*KRT74*	(6)^b^		(392)^b^
*KRT75*	9	551	*KRT75*	9	551	*KRT75*	9		549
*KRT76*	9	638	*KRT76*	9	648	*KRT76*	9		640
*KRT77*	9	578	*KRT77*	(9)^b^	(561)^b^	*KRT77*	9		593
*KRT78*	9	520	*KRT78*	9	517	*KRT78*	9		499
*KRT79*	9	535	*KRT79*	9	535	*KRT79*	9		535
*KRT80*	9	452	*KRT80*	9	453	*KRT80*	9		453
*KRT81*	9	505	*KRT81*	9	513	*KRT81*	9		507
*KRT82*	9	513	*KRT82*	9	518	*KRT82*	9		518
*KRT83*	9	493	*KRT83*	9	487	*KRT83*	9		491
*KRT84*	9	600	*KRT84*	9	586	*KRT84*	9		575
*KRT85*	9	507	*KRT85*	9	507	*KRT85*	9		507
*KRT86*	9	486	*KRT86*	9	485	*KRT86*	9		486
*KRT87P*	P^c^	P^c^	*KRT87P*	P^c^	P^c^	*KRT87P*	(8)^b^		(P)^b^^,^^c^
*KRT88P*	P^c^	P^c^	*KRT88*	(5)^b^	(153)^b^	*KRT88*	(9)^b^		(483)^b^
*KRT89P*	P^c^	P^c^	*KRT89*	9	495	*KRT89*	9		494
*KRT90P*	P^c^	P^c^	*KRT124*	(9)^b^	(531)^b^	*KRT124*		9	521
*KRT125P*	P^c^	P^c^	*-*	-	-	*-*		-	-
*KRT126P*	P^c^	P^c^	*KRT126*	9	630	*KRT126P*		(n.d.)^b^	(P)^b^^,^^c^
*KRT127P*	P^c^	P^c^	*KRT127*	9	581	*-*		-	-
*KRT128P*	P^c^	P^c^	-	-	-	*KRT128*		(10)^b^	(514)^b^
-	-	-	-	-	-	*KRT129P (LOC102149928)*		(P)^b^^,^^c^	(P)^b^^,^^c^

^a^The gene symbols have been updated by NCBI Refseq curators. A comprehensive listing of gene symbols and NCBI Gene IDs is given in S3 Table.

^b^Data in brackets are of low reliability due to gaps in the genome reference assemblies and/or insufficient coverage in the canine and equine RNA-seq data.

^c^P indicates pseudogenes.

We compiled a detailed annotation of canine and equine keratin genes in S2 Table and we provide 61 manually curated canine and equine cDNA sequences in the S1 and S2 Files.

### Keratin gene structure

The exon number of functional type I keratin genes varies between 6 and 8 in humans, dogs and horses. The length of exons 2–5 is conserved except for *KRT18*, *KRT23* and *KRT222* in all three species. The same conserved exon structure was found in *KRT42* in dog and horse but not in the human orthologous pseudogene. The length of exon 6 in keratin type I genes is 221 bp ± 3 bp except for *KRT19* and *KRT222*, which only consist of 6 exons in total and *KRT42P* in human.

**Fig 4:**
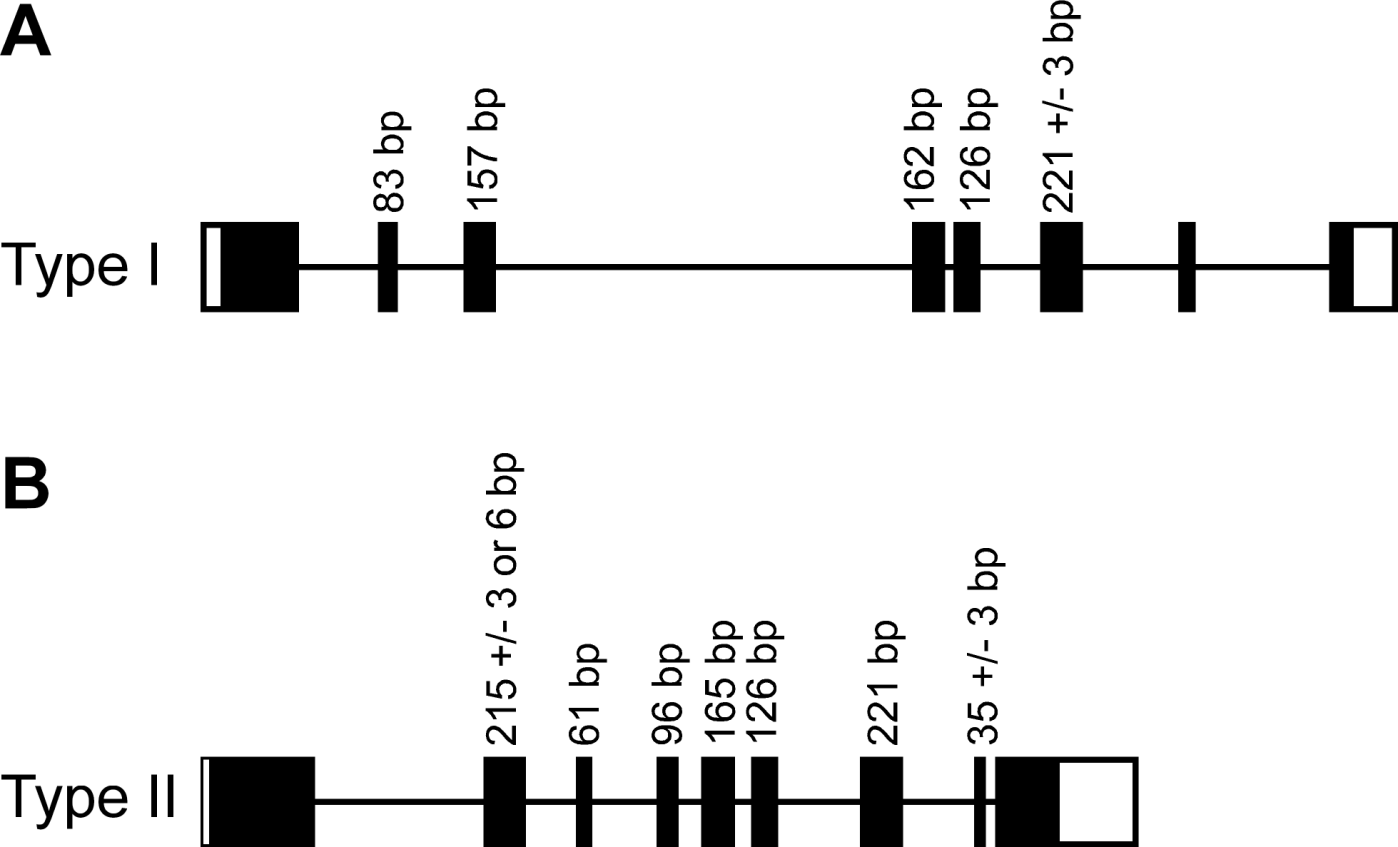
Keratin gene structure. (A) Typical type I keratin gene with eight exons. (B) Typical type II keratin gene with nine exons. The length of conserved exons is given in bp. Untranslated regions are shown as open rectangles.

Type II keratin genes in humans, dogs and horses generally have nine translated exons, except *KRT18*, *KRT74*, *KRT128*, and the canine and equine *KRT88*. The length of exons 3–7 in the type II keratin genes is conserved in all three species with the exception of *KRT8*, *KRT76*, and *KRT74*. The lengths of exons 2 and 8 varied slightly between type II keratin genes (± 3 or 6 bp). Fig 4 illustrates the typical exon/intron organization of keratin genes and S2 Table summarizes the details of all individual exons.

### Selected individual keratin genes

During our analyses, we recognized that coding exons of the equine *KRT78* and *KRT85* genes were located in gaps of the reference genome assembly (chr6:69,933,880–69,934,077 and chr6:69,459,932–69,460,612). We performed targeted sequencing of these gaps to provide the correct sequences for exon 4 of *KRT78* (96 bp, accession LT576419) and exon 7 of *KRT85* (221 bp, accession LT576418).

The annotation of canine *KRT77* was not curated due to gaps (chr27:2,412,265–2,412,884) in the reference sequence. The equine *KRT2C* gene also contains a gap probably harboring parts of coding exons (chr6:69,774,035–69,776,390). We provide a partial annotation with corrected exon numbers and inserted the presumed number of missing nucleotides to the compilation of FASTA sequences.

### Curated keratin annotations

Visual comparison of RNA-seq reads generated from skin of adult dogs and horses with our tentative annotation provided experimental support for 56 out of 62 investigated canine and 47 out of 67 investigated equine keratin genes (S2 Table). For six canine and 20 equine keratin genes, the coverage of the RNA-Seq data was insufficient for a reliable confirmation or curation/revision of the existing annotations.

### Comparison of curated keratin annotation with NCBI and Ensembl annotation

We compared our annotation of keratin genes with experimental support to the annotations from NCBI (dog annotation release 103 and horse annotation release 101) and Ensembl (release 87). Roughly half (24 of 51) canine keratin gene annotations were identical between NCBI, Ensembl and our catalog. However, 21 annotations of our catalog were only identical to the NCBI predictions but differed from Ensembl predictions. For the equine keratin genes 21 of our annotated genes were identical with Ensembl and NCBI. Of the remaining equine gene models, 18 were identical with only NCBI annotations and one was only identical to the Ensembl annotation. Five of our canine and eight of our equine curated keratin gene annotations were different from both the NCBI and Ensembl annotation.

### Updated NCBI annotation

As a result of updated NCBI annotation via NCBI’s Eukaryotic Genome Annotation Pipeline, three dog keratin genes (*KRT4*, *KRT75* and *KRT76*), which were different from our curated annotation in the previous NCBI annotation, are now identical to our annotation. Known (curated) RefSeqs replace model RefSeqs for two of these genes (*KRT4* and *KRT76*). Updates were also made following manual review of the current NCBI annotation of dog and horse keratin genes. These include gene name updates for 21 dog keratin genes and 34 horse keratin genes. Additionally, known RefSeqs replaced model RefSeqs for 19 dog keratin genes and 33 horse keratin genes (S1 Table). The gene type was updated to ‘pseudogene’ for three horse keratin genes (*KRT87P*, *KRT126P* and *KRT9P*). In the current NCBI annotation, representation of five dog keratin genes and nine horse keratin genes remain different from our curated annotation (S5 Table). However, when excluding curated annotations of low reliability due to insufficient read coverage in the RNA-seq data and keratin genes where no Ensembl annotation is given, only two canine (*KRT126* and *KRT127*) and six equine (*KRT8*, *KRT18*, *KRT16*, *KRT29*, *KRT78*, *KRT85*) keratin gene annotations differ between the updated NCBI annotation and our curated annotation.

## Discussion

We present a curated catalog of both canine and equine keratin genes, which is based on evolutionary conserved features of keratin genes and experimental support from RNA-seq data derived from adult skin. While initial comparison of our curated annotation of dog and horse keratin genes to NCBI annotation revealed several differences, the updated NCBI annotation led to prediction of several new models that were identical to our annotation (S1 Table). Additionally, a manual review of NCBI annotation by RefSeq curators resulted in several updates to gene annotation and gene nomenclature. Differences persist in the annotation of five dog and nine horse keratin genes. Many of the errors in the currently existing keratin gene predictions were due to gaps and errors in the genome reference assemblies. Although automated gene predictions in general reach a very high quality and are essential for scientists to make use of the wealth of publicly available genomic sequence information, this study also shows some of the limitations of automated gene predictions on imperfect genome assemblies.

The most striking example in our study was the *KRT9* gene. Human keratin 9 is mainly expressed in the skin at the soles of feet and the palms of hands, the so-called palmoplantar epidermis. Palmoplantar epidermis needs to withstand the highest mechanical stress on the body and thus has a specialized keratin composition. Keratin 9 is one of the key proteins that provide this extraordinary mechanical strength. Consequently, genetic variants in the *KRT9* gene can lead to palmoplantar keratoderma (OMIM ID:#144200) resulting in the localized fissuring of the palmoplantar epidermis [19,33]. The *KRT9* gene is well conserved between humans and dogs (76.1% identity within the coding sequence of the mRNA). Dogs also have a strong palmoplantar epidermis at their footpads. However, the equine ortholog of the *KRT9* gene contains a 1-nucleotide deletion in exon 6, which we assume causes the functional inactivation of this gene. Automated gene prediction tools infer information from other species and must tolerate sequencing errors, which are inherent to draft genome assemblies. In order to maintain the conserved reading frame this resulted in an NCBI transcript prediction containing an extra nucleotide, which is not present in the genome reference (XM_005614759.1). We now have experimental evidence in the form of Illumina whole genome sequence and RNA-seq data, which both confirm that in this case the equine reference genome assembly was correct and that there is indeed the 1-nucleotide deletion in the equine *KRT9P* pseudogene. In the most recent NCBI annotation release 102, XM_005614759.1 has been since replaced by XM_014736779.1 and the locus type has been updated to pseudogene as a result.

From a biological point of view, it is tempting to speculate that the extreme specialization of the equine epidermis resulting in hoof wall formation and the lack of a palmoplantar epidermis in horses might have led to decreased selection pressure on *KRT9* and eventually the fixation of the frameshift variant. This frameshift deletion in the *KRT9P* pseudogene is present in several equid species (*E*. *caballus*, *E*. *przewalski*, *E*. *asinus*), while the conserved reading frame is maintained in other ungulates such as cattle, goats, and even the perissodactyl southern rhinoceros (S2 Fig).

We think that our curated annotation of canine and equine keratin genes provides a considerable improvement with respect to the currently existing gene predictions. However, we have to caution that we also had to rely on imperfect genome reference assemblies and limited RNA-seq data, mostly restricted to adult skin. Future annotation efforts might benefit from analyzing RNA-seq data of a more comprehensive collection of tissue types and/or developmental stages. Therefore, we certainly do not claim that our version is perfect and definitive. Further research efforts will be necessary to eventually produce the complete annotation of the vastly complex keratin gene family in dogs and horses.

## Conclusions

The study of keratin genes in dog and horse is difficult because of the high duplication rate among the genes which can lead to misassemblies in the reference genomes. The manual annotation of keratin gene clusters enabled not only identification of new pseudogenes in the keratin clusters of both dog and horse, but also the identification of missing or functional canine and equine homologs of human pseudogenes. We provide a curated annotation and representative transcript sequences for canine and equine keratin genes. Correct transcript sequences are essential for many modern high-throughput technologies that rely on the bioinformatic processing of large datasets. This analysis also shows that manual intervention is still critical for the annotation of complex gene families and the production of a good reference gene set for these gene families. This work represents collaboration between a research group and a public annotation database, which resulted in improvement of annotation of a gene family in the dog and horse genomes. Such collaborations provide an opportunity for researchers and annotation groups to work together to represent specific genes or gene families that may not be accurately annotated solely by means of an automated pipeline. The updated NCBI Refseq entries will be incorporated as supporting evidence during future annotation releases by Ensembl. Many instances of low confidence annotation are in regions where the reference assembly is of poor quality, underlining the importance of a high-quality genome assembly as a prerequisite for accurate annotation of genes.

## Supporting information

S1 Fig Comparative analysis of the *KRT10* genome region. **(A)** Dot plot of the human region containing the *KRT28*, *KRT10*, and *KRT12* genes (chr17:40,794,137–40,872,876) against the corresponding dog region (chr9:21,823,976–21,910,085). Human and dog showed a well conserved synteny in this region. **(B)** Dot plot of the human region against the horse region (chr11:21,764,167–21,862,851). In the horse, a duplication event gave rise to *KRT10A* and *KRT10B* paralogs. **(C)** The horse-specific duplication also became apparent in the horse vs dog dot plot. Dot plots were generated with a word size of 10 and the software GEPARD. (PDF)
(PDF)

S2 Fig Multispecies alignment of exon 5 of the human *KRT9* gene. **(A)** This exon has a conserved length of 126 nucleotides (42 codons) in many mammalian species with high quality genome reference assemblies. It corresponds to exon 6 of the equine *KRT9P* pseudogene. In the horse, the Przewalski horse, and the donkey, the gene contains a 1 nt deletion (highlighted in grey) that leads to a frameshift and an early premature stop codon (highlighted in yellow), which truncates ~40% of the conserved open reading frame. As this frameshift deletion occurs in several equid species, it most likely arose during the early evolution of the equid family. The accessions and coordinates of the genomic sequences are given beneath the alignment. **(B)** Multispecies alignment of the translated amino acid sequences in one letter abbreviations. (PDF)
(PDF)

S3 Fig Comparative analysis of the *KRT2* genome region. **(A)** Dot plot of the human region containing the *KRT73*, *KRT128P*, *KRT2*, and *KRT1* genes (chr12:52,607,570–52,680,407) against the corresponding dog region (chr27:2,422,150–2,488,436). Human and dog showed a well conserved synteny in this region. **(B)** Dot plot of the human region against the horse region (chr6:69,698,571–69,796,491). In the horse, several duplication events gave rise to the *KRT2A*, *KRT2B*, and *KRT2C* paralogs. The support for the functional status of the equine *KRT2C* and *KRT128* genes was weak and their annotations should be considered of low confidence. **(C)** The horse-specific amplification also became apparent in the horse vs dog dot plot. Dot plots were generated with a word size of 10 and the software GEPARD. (PDF)
(PDF)

S1 File FASTA-file containing 60 curated canine keratin transcript sequences. The file is lacking a sequence for canine *KRT77* due to the low reliability of the current annotation.
(TXT)

S2 File FASTA-file containing 61 curated equine keratin transcript sequences. The file is lacking a sequence for equine *KRT74* due to the low reliability of the current annotation.
(TXT)

S3 File NIH publishing agreement & manuscript *cover sheet*. (PDF)

S1 Table Compilation of RefSeqs for canine and equine keratin genes from annotation release 104 for dogs and 102 for horses. (XLSX)

S2 Table Comparative curated annotation of human, canine and equine keratin genes. (XLSX)

S3 Table Nomenclature (gene symbols) of human, canine and equine keratin genes. (XLSX)

S4 Table Summary of keratins encoded by genes that appear to be functional in dogs or horses, but inactivated or absent in the human genome. (XLSX)

S5 Table Genes with remaining differences between NCBI annotation and our manual curation. (XLSX)
