# Supplementary figures and images for: A curated catalog of canine and equine keratin genes
Source: PLoS One. 2017 Aug 28;12(8):e0180359. doi: 10.1371/journal.pone.0180359 (PMC5573215; doi:10.1371/journal.pone.0180359)

**A**

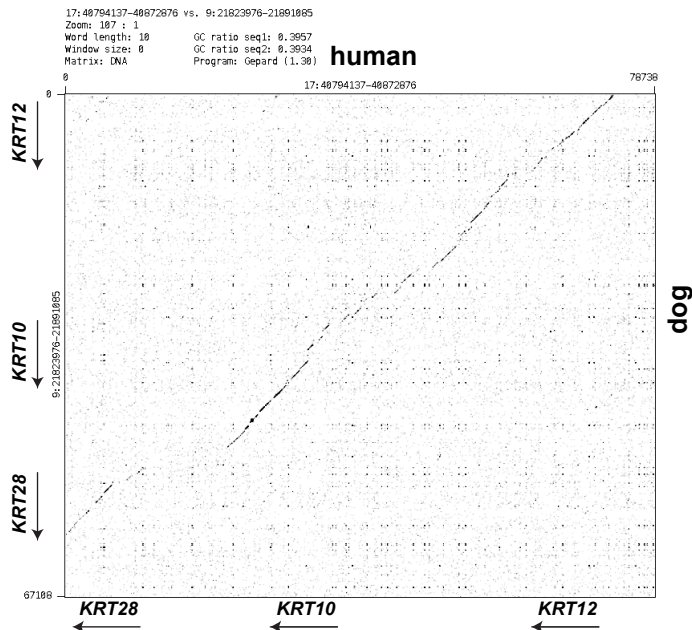

**B**

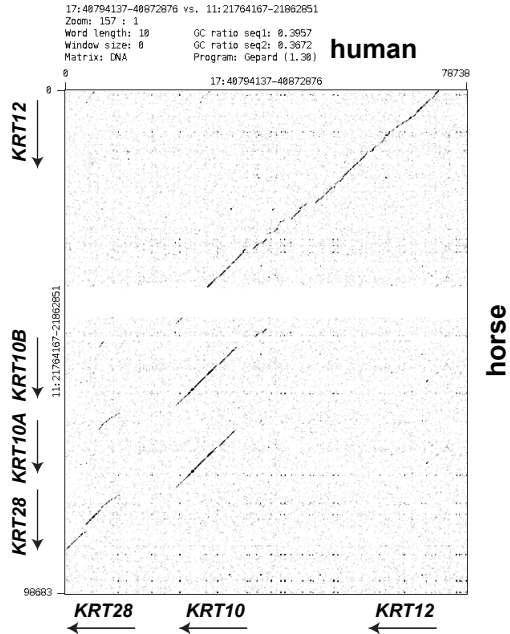

**C**

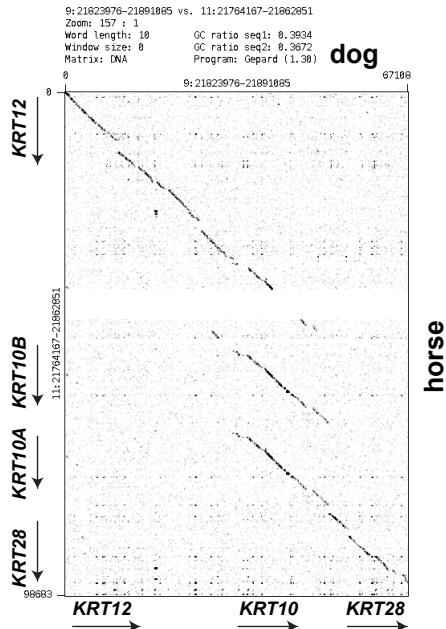

Supplement: S1 Fig — (A) Dot plot of the human region containing the KRT28, KRT10, and KRT12 genes (chr17:40,794,137–40,872,876) against the corresponding dog region (chr9:21,823,976–21,910,085). Human and dog showed a well conserved synteny in this region. (B) Dot plot of the human region against the horse region (chr11:21,764,167–21,862,851). In the horse, a duplication event gave rise to KRT10A and KRT10B paralogs. (C) The horse-specific duplication also became apparent in the horse vs dog dot plot. Dot plots were generated with a word size of 10 and the software GEPARD. (PDF) (PDF) [file pone.0180359.s001.pdf]

**A**

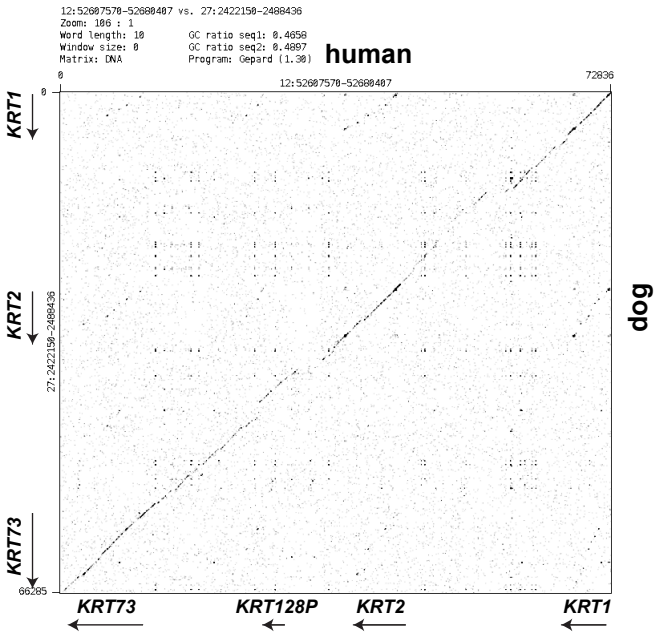

**B**

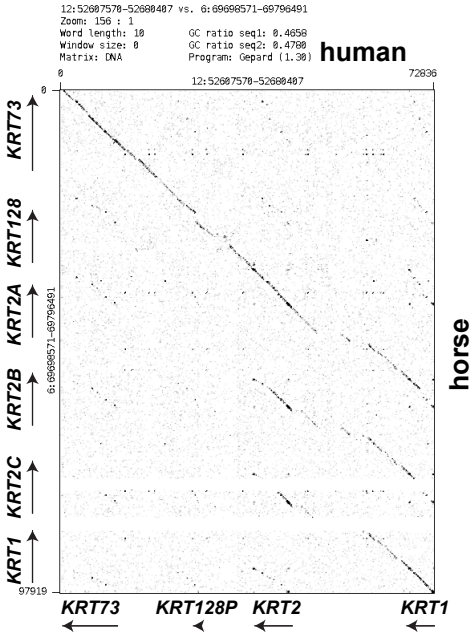

**C**

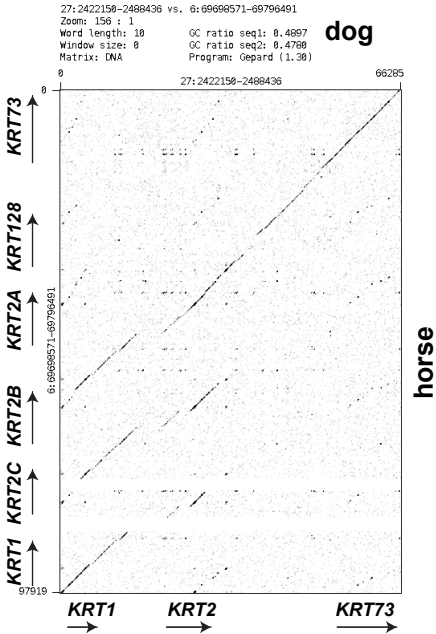

Supplement: S3 Fig — (A) Dot plot of the human region containing the KRT73, KRT128P, KRT2, and KRT1 genes (chr12:52,607,570–52,680,407) against the corresponding dog region (chr27:2,422,150–2,488,436). Human and dog showed a well conserved synteny in this region. (B) Dot plot of the human region against the horse region (chr6:69,698,571–69,796,491). In the horse, several duplication events gave rise to the KRT2A, KRT2B, and KRT2C paralogs. The support for the functional status of the equine KRT2C and KRT128 genes was weak and their annotations should be considered of low confidence. (C) The horse-specific amplification also became apparent in the horse vs dog dot plot. Dot plots were generated with a word size of 10 and the software GEPARD. (PDF) (PDF) [file pone.0180359.s003.pdf]
