# Supplementary material for: A curated catalog of canine and equine keratin genes
Source: PLoS One. 2017 Aug 28;12(8):e0180359. doi: 10.1371/journal.pone.0180359 (PMC5573215; doi:10.1371/journal.pone.0180359)
Supplement: S2 Fig — (A) This exon has a conserved length of 126 nucleotides (42 codons) in many mammalian species with high quality genome reference assemblies. It corresponds to exon 6 of the equine KRT9P pseudogene. In the horse, the Przewalski horse, and the donkey, the gene contains a 1 nt deletion (highlighted in grey) that leads to a frameshift and an early premature stop codon (highlighted in yellow), which truncates ~40% of the conserved open reading frame. As this frameshift deletion occurs in several equid species, it most likely arose during the early evolution of the equid family. The accessions and coordinates of the genomic sequences are given beneath the alignment. (B) Multispecies alignment of the translated amino acid sequences in one letter abbreviations. (PDF) (PDF) [file pone.0180359.s002.pdf]

A

```
Homo sapiens          IleThrGlnIleGluHisGluValSerSerSerGlyGlnGluValGlnSerSerAlaLysGluValThrGlnLeuArgHisGlyValGlnGluLeuGluIleGluLeuGlnSerGlnLeuSerLys
Homo sapiens        agATAACCCAGATCGAGCATGAGGTATCCAGTAGTGGTCAGGAGGTGCAGTCCAGTGCCAAGGAGGTGACCCAGCTCCGGCACGGTGTCCAGGAGTTGGAGATTGAGCTGCAGTCTCAGCTCAGCAAGgt

Equus caballus        MetSerGlnIleGluGlnGluValThrAsnArgSerGlnGluMetGluLeuAsnAsnA:rgArgTER
Equus caballus      agATGAGCCAGATCGAGCAGGAGGTGACGAATAGAAGCCAAGAAATGGAGCTCAACAACA:GGAGGTGAGCCAGCTCCGGCACAGCATCCAGGAGTTGGAGATGGAGCTGCAGTCTCAGCTCAGCACGgt

Equus przewalski      MetSerGlnIleGluGlnGluValThrAsnArgSerGlnGluMetGluLeuAsnAsnA:rgArgTER
Equus przewalski    agATGAGCCAGATCGAGCAGGAGGTGACGAATAGAAGCCAAGAAATGGAGCTCAACAACA:GGAGGTGAGCCAGCTCCGGCACAGCATCCAGGAGTTGGAGATGGAGCTGCAGTCTCAGCTCAGCACGgt

Equus asinus          MetSerGlnIleGluGlnGluValThrAsnArgSerGlnGluMetGluLeuAsnAsnA:rgArgTER
Equus asinus        agATGAGCCAGATCGAGCAGGAGGTGACGAATAGAAGCCAAGAAATGGAGCTCAACAACA:GGAGATGAGCCAGCTCCGGCACAGCATCCAGGAGTTGGAGATGGAGCTGCAGTCTCAGCTCAGCACGgt

Ceratotherium simum   MetSerGlnIleGluGlnGluValThrAsnArgSerGlnGluMetGluPheAsnAsnLysGluGluThrGlnHisArgHisSerIleGlnGluLeuGluMetGluLeuGlnSerGlnLeuSerThr
Ceratotherium simum agATGAGCCAGATTGAGCAGGAGGTAACAAACAGAAGCCAGGAAATGGAGTTCAACAACAAAGAAGAGACCCAGCACCGGCATAGCATCCAGGAGTTGGAGATGGAGCTGCAGTCTCAGCTCAGTACGgt

Capra hircus          MetLysGlnIleGluGlnGluValThrThrCysSerGlnGluValGluSerSerAsnLysGluValThrLysLeuArgHisThrValGlnGluLeuGluValGluLeuGlnSerGlnPheSerMet
Capra hircus        agATGAAACAGATTGAGCAAGAAGTGACGACCTGTAGCCAGGAGGTGGAGTCCAGCAACAAGGAGGTGACCAAGCTTCGGCACACCGTCCAAGAATTGGAGGTCGAGCTGCAGTCTCAGTTCAGCATGgt

Canis familiaris      MetSerGlnMetGluGlnGluValMetSerSerGlyGlnGluMetGluSerAsnHisLysGluValThrGlnLeuArgHisSerIleGlnGluMetGluIleGluLeuGlnSerGlnLeuSerLys
Canis familiaris    agATGAGCCAAATGGAGCAGGAAGTGATGAGTAGTGGCCAGGAGATGGAGTCCAACCACAAGGAGGTGACCCAGCTCCGGCACAGCATCCAGGAGATGGAGATTGAGCTGCAGTCTCAGCTCAGCAAGgt

Mus musculus          MetThrGlnIleGluGlnGlnMetThrAsnSerGlyGlnGluMetGluSerAsnMetLysGlnValSerGlnLeuGlnHisThrIleGlnGluLeuAsnValGluLeuGlnThrGlnLeuThrThr
Mus musculus        agATGACCCAGATTGAGCAGCAAATGACAAATAGTGGCCAAGAGATGGAGAGCAACATGAAGCAAGTGTCCCAGCTCCAGCACACTATCCAGGAACTGAATGTGGAGCTGCAGACTCAGCTTACCACGgt
```

Homo sapiens (human)	GRCh38/hg38	chr17:41,568,506-41,568,635	(rev.-compl.)		ITQIEHEVSSSGQEVQSSAKEVTQLRHGVQELEIELQSQLSK
Equus caballus (horse)	EquCab 2	chr11:21,215,793-21,215,854			MS...Q..TNRS..MELNNRR*
Equus przewalski (Przewalski horse)	Burgud	NW_007676318.1:164,473-164,601			MS...Q..TNRS..MELNNRR*
Equus asinus (donkey)	ASM130575v1	NW_014638702.1:10,952,197-10,952,325			MS...Q..TNRS..MELNNRR*
Ceratotherium simum (white rhino)	CerSim 1	JH767772:1,244,645-1,244,774			MS...Q..TNRS..MEFNN..E..H..SI....M.......T
Capra hircus (goat)	ASM170441v1	chr19:41,425,490-41,425,619	(rev.-compl.)		MK...Q..TTCS...E..N....K...T.....V.....F.M
Canis familiaris (dog)	CanFam3.1	chr9:21,207,910-21,208,035			MS.M.Q..M.....ME.NH........SI..M..........
Mus musculus (mouse)	GRCm38/mm10	chr11:100,189,919-100,190,048	(rev.-compl.)	**B**	M....QQMTN....ME.NM.Q.S..Q.TI...NV...T..TT
